# Benefits of home-based foot neuromuscular electrical stimulation on self-reported function, leg pain and other leg symptoms among community-dwelling older adults: a sham-controlled randomised clinical trial

**DOI:** 10.1186/s12877-024-05271-z

**Published:** 2024-08-14

**Authors:** Binoy Kumaran, Darren Targett, Tim Watson

**Affiliations:** 1 School of Health and Social Work, University of Hertfordshire, Hatfield, AL10 9AB UK; 2 Primoris Contract Solutions Ltd, Ascot, SL5 0LW UK

**Keywords:** Neuromuscular electrical stimulation (NMES), Blood flow, Leg pain, Leg symptoms, Self-reported function

## Abstract

**Introduction:**

Lower leg pain and symptoms, and poor leg circulation are common in older adults. These can significantly affect their function and quality of life. Neuromuscular electrical stimulation (NMES) applied via the feet as ‘foot NMES’ activates the leg musculovenous pump. This study investigated the effects of foot NMES administered at home using Revitive^®^ among community-dwelling older adults with lower leg pain and/or other lower leg symptoms such as cramps, or sensations of tired, aching, and heavy feeling legs.

**Methods:**

A randomised placebo-controlled study with three groups (2 NMES, 1 Sham) and three assessments (baseline, week 8, week 12 follow-up) was carried out. Self-reported function using Canadian occupational performance measure (COPM), leg pain, overall leg symptoms score (heaviness, tiredness, aching, or cramps), and ankle blood flow were assessed. Analysis of covariance (ANCOVA) and logistic regression were used to compare the groups. Statistical significance was set at *p* < 0.05 (two-sided 5%).

**Results:**

Out of 129 participants enrolled, 114 completed the study. The improvement in all outcomes were statistically significant for the NMES interventions compared to Sham at both week 8 (*p* < 0.01) and week 12 (*p* < 0.05). The improvement in COPM met the minimal clinically important difference (MCID) for the NMES interventions compared to Sham at both week 8 (*p* < 0.005) and week 12 (*p* < 0.05). Improvement in leg pain met MCID at week 8 compared to Sham (*p* < 0.05). Ankle blood flow increased approximately 3-fold during treatment compared to Sham. Compliance with the interventions was high and no device-related adverse events were reported.

**Conclusions:**

The home-based foot NMES is safe, and significantly improved self-reported function, leg pain and overall leg symptoms, and increased ankle blood flow compared to a Sham among older adults.

**Trial registration:**

The trial was prospectively registered in ISRCTN on 17/06/2019 with registration number ISRCTN10576209. It can be accessed at https://www.isrctn.com/ISRCTN10576209.

**Supplementary Information:**

The online version contains supplementary material available at 10.1186/s12877-024-05271-z.

## Key points

• Eight weeks of daily home-based foot NMES therapy delivered using Revitive devices significantly improved self-reported function, leg pain and other leg symptoms in community-dwelling older adults when compared to a Sham.

• Significant improvements in self-reported function and leg symptoms were sustained when measured at the follow-up four weeks after the intervention period.

• During use, the foot NMES induced approximately a three-fold increase in ankle blood flow volume and intensity of blood flow when compared to a Sham.

• No device-related adverse events were reported.

• Compliance with the foot NMES intervention was high.

**Supplementary Information:**

The online version contains supplementary material available at 10.1186/s12877-024-05271-z.

## Introduction

‘Healthy ageing’ is vital as the world population is becoming older. As life expectancy increases an active lifestyle is key to maintaining functional ability and to promote healthy ageing, thereby minimising the impact on quality of life (QoL) [1, 2]. Lower leg symptoms such as pain and cramps, and sensations of tired, aching, and heavy feeling legs are common in older adults and can significantly impact on their functional QoL [3–8]. It is often difficult to associate the causes of such symptoms to a specific diagnosis, especially when multiple comorbidities relating to different medical conditions are present. Functional limitations due to ageing, a sedentary lifestyle or underlying comorbidities (e.g. peripheral vascular disease (PVD)) can perpetuate a ‘vicious circle’ leading to a more sedentary life, increased leg symptoms and a further decline in function [3–5, 9, 10].

Reduced lower leg muscle strength is a predictor of disability among older adults [11, 12]. Alongside poor blood flow, due to deterioration of the calf muscle pump, weakness in the legs is a common problem seen among older adults [11, 12]. Neuromuscular electrical stimulation (NMES) is a widely used modality for muscle strengthening and functional rehabilitation across various clinical populations, with a significant body of supporting literature [13–17]. Additionally, NMES treatment can boost blood circulation in asymptomatic people [18, 19], as well as in those with underlying circulatory deficits [20–24]. Instead of delivering through the conventional body pads, when NMES is applied as a novel ‘foot NMES’ via the plantar surface, the induced muscle contractions activate the musculovenous pump thus stimulating blood flow during treatment [19]. Whilst improved blood flow helps maintain leg circulatory health, muscle contractions can help improve strength and mobility [18, 25–31], demonstrating clinical benefits in patients with PVDs such as chronic venous disease (CVD) and peripheral arterial disease (PAD) by relieving symptoms, and improving functional QoL [32–34].

There is a paucity of research investigating the effects of home-based foot NMES on leg pain and other leg symptoms, and the functional QoL among community-dwelling older adults. Contemporary research lacks sham-controlled studies and are focused mainly on clinician delivered NMES with various patient groups despite home-based care becoming increasingly important for reducing the pressures on healthcare providers [35–37]. Therefore, the objective of this study was to investigate the effects of an eight-week foot NMES treatment program administered at home using two different Revitive^®^ devices on older adults with leg pain and/or leg symptoms and to compare them to a Sham. The study protocol has been published [38].

## Methods

This study is reported in accordance with the Consolidated Standards of Reporting Trials (CONSORT) guidelines [39].

### Study design

This study investigated the effects of two different foot NMES programmes against a Sham. It was a single-centre (Physiotherapy research laboratory at University of Hertfordshire, Hatfield, UK), participant-blind, parallel-group, randomised, placebo-controlled (Sham group), interventional study of 12-week duration (8-week intervention, and 4-week follow-up) with three assessments (baseline, week 8, and week 12) and three groups, each receiving a different type of foot NMES:

- Group 1 – Revitive®Sham.
- Group 2 – Revitive®Medic^©^ Program 1.
- Group 3 – Revitive®Program 2.

**Fig. 1:**
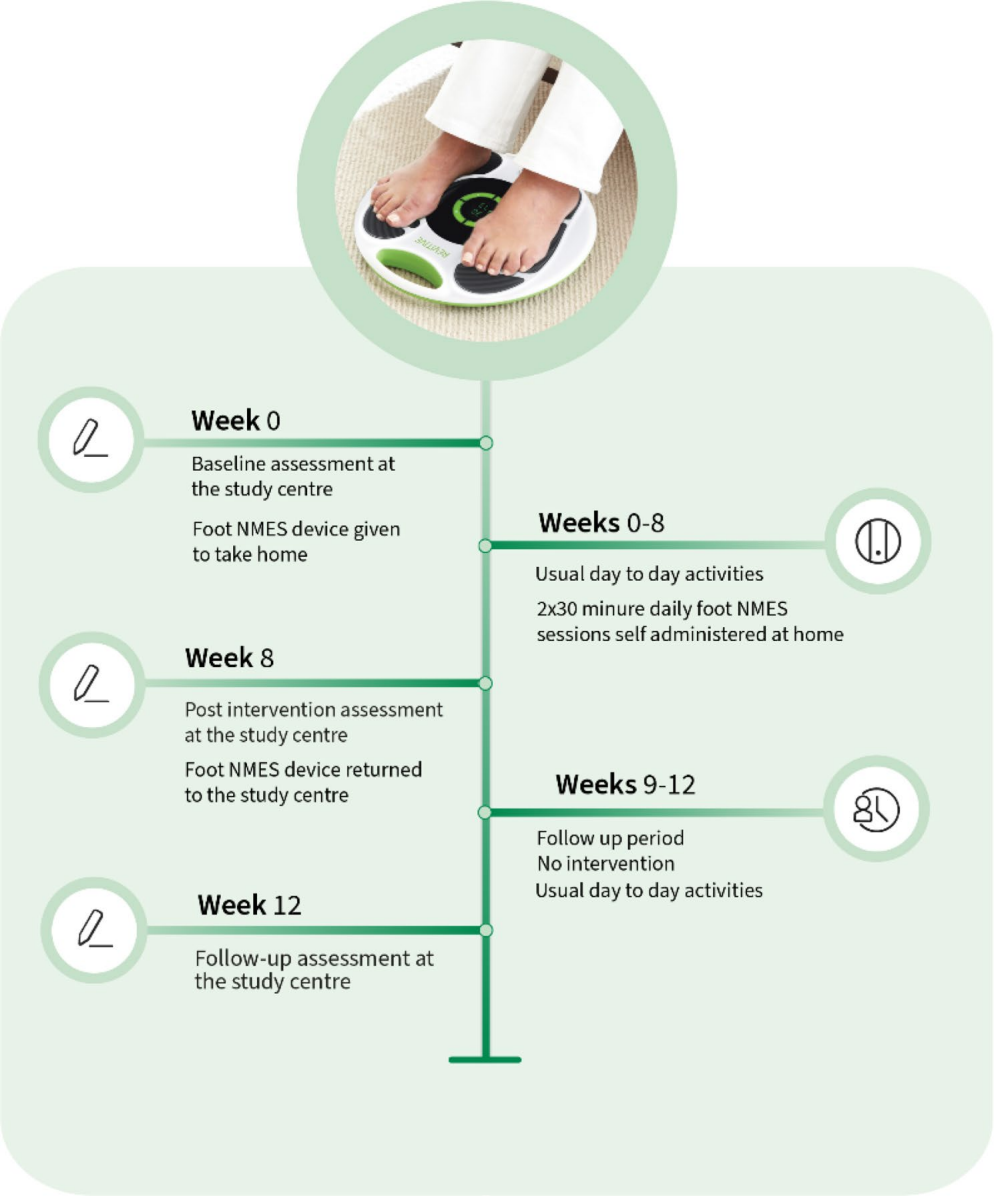
Revitive^®^ device and the study structure

An overview of the study structure is given in Fig. 1. More details can be found in the published study protocol [38].

### Participants

The study was advertised to the public using multiple local newspapers. Participants were community-dwelling adults aged over 65 years who reported one or more of the following symptoms in one or both legs: heaviness, tiredness, aching, or cramps. Exclusion criteria (self-reported) included: severe diabetic neuropathy; lumbar radiculopathy; restless legs syndrome; nervous system disorders; active cancer; contra-indications to NMES such as implanted electronic device (e.g., cardiac pacemaker); significant recent injury to the leg(s) (within the last six months); symptoms related to autoimmune, rheumatological, systemic illnesses or musculoskeletal conditions; those currently using Revitive^®^; being non-ambulant; inability to communicate in English; and inability to provide informed consent. The participants did not need to present with any specific diagnosis and the recruitment was solely based on self-reported symptoms.

### Randomisation and blinding

Participants were randomised in a 1:1:1 ratio using computer-generated blocks of nine generated by the third author. The first author (principal investigator, PI) enrolled and assigned the participants to the interventions. Allocation was blinded by concealment of the randomisation schedule. All three Revitive^®^ devices (Fig. 1) and their user manuals were identical. Conducting a double-blind study was problematic, as the assessor could identify the intervention groups during blood flow measurements due to visible muscle contractions (unlike for Sham). The participants remained blinded throughout the study. All data were processed and analysed by an independent statistician (second author).

### Intervention

The study investigated the effects of two different Revitive^®^ foot NMES programmes, against a Sham. The devices featured a mechanical foot rocker function (patented IsoRocker). Revitive^®^ Medic© Program 1 comprised 15 NMES waveforms (patented). Revitive^®^ Program 2 comprised 6 NMES waveforms (patent pending). The stimulation intensity was variable (1 to 99) and was controlled by the user. The comparator was Revitive^®^ Sham, which was identical to Program 1, except that the stimulation intensity was limited to ‘2’ (delivered in 99 increments). The Sham devices delivered a weak yet perceivable sensation thus promoting a placebo effect among the recipients in that group rather than delivering a ‘no treatment’ control intervention. All devices were timed to run continuously for 30 min. Participants completed two 30-minute sessions at home daily for eight weeks, with the mechanical foot rocker enabled. They were advised to maintain a strong but comfortable stimulation intensity. Participants were told that the perception of stimulation may vary between people or may not be felt at all, and that the sensation often becomes less noticeable over time. All participants were supported throughout the study for any technical or clinical concerns.

### NMES parameters

The Revitive^®^ foot NMES devices used in this study comprise biphasic waveforms with frequencies ranging from 20 to 43.7 Hz. The pulse durations of the waveforms range from 450 to 970 µs. The maximum current output of the devices is 15 mA RMS at 500Ω resistance. The ‘ON’ and ‘OFF’ duration of NMES is waveform dependent and ranges from 1.9 to 8.3 s and 1.0 to 1.5 s respectively.

### Primary outcome

The primary outcome was change in the Canadian Occupational Performance Measure Performance (COPM-P) from baseline to Week 8 for Revitive^®^ Medic© Program 1 versus Sham. The COPM-P is a self-evaluation measure of each participant’s current physical/functional performance on self-selected activities. The COPM [40] is a valid, reliable, and responsive outcome measure widely used in clinical research among older adults [41–43]. The COPM was administered by the investigator in an open dialogue with the participants during their study visits. The COPM functional activities most commonly reported by the participants in this study were sleeping, walking, sitting, standing, and stair climbing.

### Secondary outcomes

The secondary outcomes were COPM satisfaction (COPM-S), which measures the individual’s satisfaction with their COPM-P, leg pain measured using numerical pain rating scale (NPRS), total daily overall leg symptoms score [44] (the number of symptom days multiplied by the average intensity, summed across all four symptom domains: heaviness, tiredness, aching, and cramps), and deep ankle blood flow volume and intensity of flow measured using Doppler ultrasound before and during NMES application. Like the primary outcome, all secondary outcomes were administered by the same investigator (first author) during the study visits. Blood flow was recorded (first author is trained in ultrasound blood flow measurements) with the mechanical rocker disabled (to reduce noise), using ‘Esaote MyLab70 XVG’ ultrasound scanner with ‘LA523’ probe (4–13 MHz, Esaote S.p.A, Genoa, Italy).

All outcomes except blood flow were assessed at weeks 0, 8, and 12. Blood flow was recorded before and during NMES application at Week 0 only as it was a spot measurement unlike the other outcomes, and based on previous pilot experiments, the authors did not expect the relative change in blood flow during the NMES application to change over the study duration.

### Adverse events monitoring

The Revitive NMES is a commercially available over the counter (OTC) device used extensively in the community. The risk of serious adverse events (SAE) is very rare. The occurrence of all adverse events (AE), whether or not device-related, was recorded throughout this study. Participants were instructed to report all AEs to the investigator as soon as possible. They were also asked about the occurrence of such events at study visits. AEs of special interest were identified as events with a potential causal association to the use of the study device. Any SAEs were investigated by an independent medical monitor and reported to the Ethics Committee and regulatory authorities.

### Compliance monitoring

The participants were instructed to keep a daily record of treatment sessions undertaken. In the rare event that one or more sessions is missed, participants were required to keep a written record of those events and report them to the investigator at the Week 8 visit. The investigator documented the total number of sessions missed by each participant during their intervention period. All data were included in the statistical analysis.

### Statistical analysis

Statistical analyses were conducted (using SAS version 9.4) for intent-to-treat (ITT), modified intent-to-treat (MITT) and per protocol (PP) populations; MITT and PP analyses were considered secondary. The MITT population included all participants who used their intervention at least once (ITT population), and if the condition being assessed was present at baseline. The PP population included participants who demonstrated a minimum 75% compliance with the intervention (missing no more than 28 treatments out of 112 over eight weeks). Missing data were imputed using multiple imputation under a ‘missing at random’ assumption using a monotone regression model.

Analysis of covariance (ANCOVA) with baseline value as a covariate and treatment group as a classification variable was used to compare Program 1 versus Sham and Program 2 versus Sham at weeks 8 and 12. If model assumptions were not met, Wilcoxon rank sum test was used. To control for multiplicity, a hierarchical testing procedure was used, whereby the statistical significance of Program 1 versus Sham was evaluated, and if this achieved significance (*p* < 0.05), the comparison of Program 2 versus Sham was evaluated. Logistic regression was used to compare the proportion of ‘responders’ (participants who achieved a minimal clinically important difference (MCID) in the outcome: 2-point improvements in COPM and leg pain) in each test group versus Sham. Treatment effect was estimated as an odds ratio (test/Sham), with 95% CIs and p-value. An odds ratio > 1 indicated a better outcome in the test group. Ultrasound images were computationally analysed using MATLAB (MathWorks, Massachusetts) algorithms to process colour image data into numerical data prior to statistical analysis, using a published method [45].

#### Interim analysis and sample size

An interim analysis of COPM-P was conducted with the first 10 participants from each group (total 30) to confirm sample size. An improvement of two points in individual COPM-P score was considered the MCID [43, 46, 47]. An absolute difference of 30% in the proportion of participants that met the MCID between Revitive^®^ Sham, and Programs 1 or 2 was considered necessary to demonstrate a clinically meaningful difference for either active intervention [48–51]. The responder rate (participants who met the MCID) was calculated for Sham, and an absolute risk difference was defined for determining the required responder rates for Programs 1 and 2. Based on this model 39 participants were needed in each group (80% power, two-sided 5% significance, medium to large effect size of 0.643). Pearson Chi-square test at two-sided significance level (*p* < 0.05) was used for this comparison. Participant data from the interim analysis was included in the final analysis, as an identical protocol was followed throughout. No hypothesis test for stopping for futility or efficacy was conducted at the interim analysis, therefore the potential for inflation of Type I or Type II errors was considered negligible.

#### Post-hoc analysis

A post-hoc responder analysis was performed for leg pain, where an improvement by at least 30% compared to baseline was considered the MCID, instead of the conventional 2-point absolute change. The rationale for this analysis was that in studies with higher levels of variability in baseline pain (such as this study where there was no ‘minimum pain’ entry criterion), the relationship between percentage change and participant perception of improvement is more consistent than the relationship between raw change and perception of improvement [52, 53]. Therefore, the relative change provides additional and pertinent evidence.

## Results

**Fig. 2:**
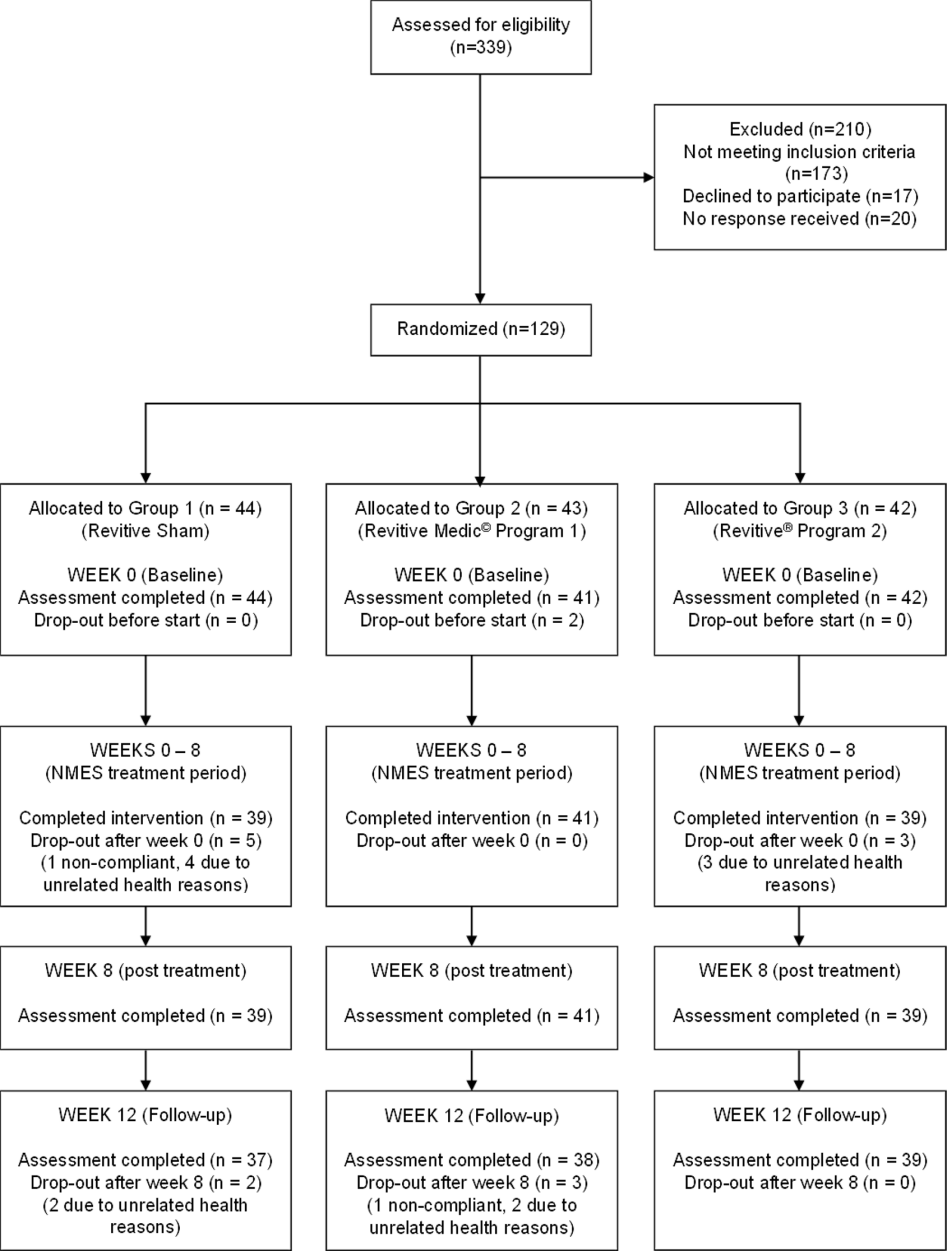
The CONSORT diagram

The study flowchart is given in Fig. 2. The demographic and anthropometric data are detailed in Table 1. There were no notable differences in these characteristics between the three treatment groups. Patient self-reported comorbidities are given in Additional Table 1.

**Table 1:** Demographic and anthropometric data

Study Group	Demographic data			Mean (SD) anthropometric data		
	Mean (SD) age (years)	Males	Females	Height (m)	Weight (kg)	BMI
Group 1 Revitive^®^ Sham	72.5 (6.38)	24	20	1.645 (0.1001)	77.26 (18.990)	28.38 (5.577)
Group 2 Revitive^®^ Medic© Program 1	72.8 (5.49)	17	24	1.626 (0.0969)	82.30 (17.594)	31.04 (5.736)
Group 3 Revitive^®^ Program 2	73.0 (5.68)	21	21	1.643 (0.0828)	77.71 (19.403)	28.56 (5.931)

### Compliance, adverse events, and dropouts

Among the 129 participants enrolled, 127 formed the ITT population, of which 119 completed Week 8 assessments (8 dropouts) and 114 completed Week 12 assessments (further 5 dropouts) (Fig. 2). Compliance with the interventions were high, with > 92% in the two test groups and > 88% in the Sham group completing at least 75% of the required number of treatments over eight weeks. Excluding ten non-compliant participants (5 from Sham, 2 from Program 1, and 3 from Program 2), 117 were analysed in the PP population. The dropouts were due to unrelated AEs (UAE) and/or due to non-compliance. No serious adverse events (SAE) were reported and none of the AEs reported were determined as having any causal relationship to the interventions. The recruitment started on 01 July 2019 and finished on 30 June 2022. The study was successfully completed on 31 October 2022.

## Main results

Tables 2 and 3 summarise the results of statistical analyses from all primary and secondary outcomes. The results of sensitivity analysis and PP analysis were consistent with the primary analyses for all instances (not reported). The mean/median percentage changes in scores from baseline for each outcome are reported in Additional Table 2.

**Table 2:** Results of COPM-P, COPM-S, overall leg symptoms score, blood flow volume, and blood flow intensity ITT analyses

Outcome measure	Time point	Statistic	ITT analysis		
			Program 1	Program 2	Sham
COPM-P	Week 8	LS Mean	2.23	2.12	1.09
		Difference to Sham	1.14	1.03	
		95% CI	0.58, 1.69	0.47, 1.59	
		*p value (vs. Sham)*	*< 0.0001*	*0.0003*	
		Responders	25 (61%)	25 (60%)	9 (21%)
		Non-responders	16 (39%)	14 (33%)	30 (68%)
		Missing	0	3 (7%)	5 (11%)
		Odds ratio	4.86	4.79	
		95% CI	1.82, 12.93	1.80, 12.74	
		*p value (vs. Sham)*	*0.0016*	*0.0017*	
	Week 12	LS Mean	2.05	1.95	0.97
		Difference to Sham	1.08	0.99	
		95% CI	0.52, 1.64	0.42, 1.56	
		*p value (vs. Sham)*	*0.0002*	*0.0007*	
		Responders	21 (51%)	25 (56%)	10 (23%)
		Non-responders	17 (42%)	14 (33%)	27 (61%)
		Missing	3 (7%)	3 (7%)	7 (16%)
		Odds ratio	3.29	4.35	
		95% CI	1.25, 8.69	1.64, 11.59	
		*p value (vs. Sham)*	*0.0163*	*0.0033*	
COPM-S	Week 8	LS Mean	2.6	2.4	1.34
		Difference to Sham	1.26	1.05	
		95% CI	0.61, 1.90	0.40, 1.71	
		*p value (vs. Sham)*	*0.0002*	*0.0016*	
		Responders	30 (73%)	27 (64%)	8 (18%)
		Non-responders	11 (27%)	12 (29%)	31 (71%)
		Missing	0	3 (7%)	5 (11%)
		Odds ratio	8.80	7.16	
		95% CI	2.95, 26.24	2.41, 21.29	
		*p value (vs. Sham)*	*< 0.0001*	*0.0004*	
	Week 12	LS Mean	2.34	2.25	1.22
		Difference to Sham	1.12	1.03	
		95% CI	0.47, 1.78	0.37, 1.68	
		*p value (vs. Sham)*	*0.0008*	*0.0021*	
		Responders	25 (61%)	25 (60%)	9 (20%)
		Non-responders	13 (32%)	14 (33%)	28 (64%)
		Missing	3 (7%)	3 (7%)	7 (16%)
		Odds ratio	4.8	4.86	
		95% CI	1.73, 13.35	1.72, 13.77	
		*p value (vs. Sham)*	*0.0027*	*0.0029*	
Symptoms score	Week 8	LS Mean	-10.17	-10.49	-5.95
		Difference to Sham	-4.21	-4.53	
		95% CI	-6.53, -1.89	-6.84, -2.22	
		*p value (vs. Sham)*	*0.0004*	*0.0001*	
	Week 12	LS Mean	-9.01	-8.95	-5.52
		Difference to Sham	-3.49	-3.42	
		95% CI	-6.22, -0.75	-6.07, -0.77	
		*p value (vs. Sham)*	*0.0125*	*0.0113*	
Blood flow volume	Pre	Mean	2.578		2.569
		SD	1.4529		1.478
	During	Mean	7.511		2.542
		SD	3.4721		1.6965
	Change	LS Mean	4.934		-0.028
		Difference to Sham	4.961		
		95% CI	3.942, 5.981		
		*p value (vs. Sham)*	*< 0.0001*		
Blood flow intensity	Pre	Mean	223.1		220.2
		SD	141.85		135.83
	During	Mean	730.1		243.9
		SD	363.24		196.17
	Change	LS Mean	507.32		23.15
		Difference to Sham	484.17		
		95% CI	372.4, 595.9		
		*p value (vs. Sham)*	*< 0.0001*		

The COPM is measured from 0 (worst) to 10 (best). Overall symptoms score ranged from 0 (least symptoms) to 40 (worst symptoms). Missing values were imputed using Multiple Imputation under a missing at random assumption. LS Mean is Least Squares Mean from ANCOVA with a factor for treatment group and baseline score as a covariate. Difference expressed as Test minus Sham such that a positive difference favours the test group for COPM and Blood Flow, and a negative difference favours the test group for Leg Symptoms Score. Responder is a participant whose COPM score improved by two or more points. Odds ratio (versus Sham) from a logistic regression model estimating the probability of being a responder with treatment group as a factor and baseline score as a covariate. An odds ratio > 1 means participants in test groups are more likely to be responders compared to those in the Sham group. For blood flow Programs 1 & 2 were combined to form one group

**Table 3:** Results of leg pain ITT, MITT and post-hoc responder analyses

Time point	Statistic	ITT analysis			MITT analysis			Post-hoc ITT analysis			Post-hoc MITT analysis		
		Program 1	Program 2	Sham	Program 1	Program 2	Sham	Program 1	Program 2	Sham	Program 1	Program 2	Sham
Week 8	LS Mean	-3.48	-3.2	-1.91	-3.92	-3.61	-2.17						
	Difference to Sham	-1.57	-1.29		-1.75	-1.44							
	95% CI	-2.51, -0.64	-2.22, -0.36		-2.80, -0.7	-2.52, -0.35							
	*p value (vs. Sham)*	*0.001*	*0.0066*		0.0011	0.0093							
	Responders	28 (68%)	28 (67%)	19 (43%)	28 (78%)	28 (76%)	19 (47%)	28 (68%)	25 (60%)	16 (36%)	28 (78%)	25 (68%)	16 (40%)
	Non-responders	13 (32%)	11 (26%)	20 (46%)	8 (22%)	6 (16%)	16 (40%)	13 (32%)	14 (33%)	23 (52%)	8 (22%)	9 (24%)	19 (48%)
	Missing	0	3 (7%)	5 (11%)	0	3 (8%)	5 (13%)	0	3 (7%)	5 (12%)	0	3 (8%)	5 (12%)
	Odds ratio	2.81	4.18		2.91	4.03		3.36	3.03		4.05	3.25	
	95% CI	0.99, 7.97	1.38, 12.66		1.04, 8.17	1.28, 12.67		1.25, 9.02	1.14, 8.04		1.45, 11.28	1.16, 9.10	
	*p value (vs. Sham)*	*0.0523*	*0.0113*		*0.0426*	*0.0174*		*0.016*	*0.0257*		*0.0075*	*0.0247*	
Week 12	LS Mean	-2.62	-2.76	-1.53	-2.95	-3.17	-1.73						
	Difference to Sham	-1.09	-1.24		-1.22	-1.44							
	95% CI	-2.09, -0.09	-2.24, -0.23		-2.33, -0.1	-2.55, -0.32							
	*p value (vs. Sham)*	*0.032*	*0.0157*		0.0329	0.0117							
	Responders	20 (49%)	24 (57%)	16 (36%)	20 (56%)	24 (65%)	16 (40%)	20 (49%)	23 (55%)	13 (29%)	20 (56%)	23 (62%)	13 (32%)
	Non-responders	18 (44%)	15 (36%)	21 (48%)	13 (36%)	10 (27%)	17 (43%)	18 (44%)	16 (38%)	24 (55%)	13 (36%)	11 (30%)	20 (50%)
	Missing	3 (7%)	3 (7%)	7 (16%)	3 (8%)	3 (8%)	7 (17%)	3 (7%)	3 (7%)	7 (16%)	3 (8%)	3 (8%)	7 (18%)
	Odds ratio	1.58	2.38		1.83	2.88		2.07	2.73		2.45	3.37	
	95% CI	0.61, 4.08	0.90, 6.32		0.68, 4.91	1.01, 8.23		0.81, 5.33	1.05, 7.13		0.89, 6.76	1.18, 9.62	
	*p value (vs. Sham)*	*0.3502*	*0.0817*		*0.2285*	*0.0494*		*0.1302*	*0.0405*		*0.0828*	*0.0234*	

Leg pain is measured from 0 (least pain) to 10 (worst pain). Missing values were imputed using Multiple Imputation under a missing at random assumption. LS Mean is Least Squares Mean from ANCOVA with a factor for treatment group and baseline score as a covariate. Difference expressed as Test minus Sham such that a negative difference favours the test group for Leg Pain. Responder for the planned analysis is a participant whose leg pain score improved by two or more points. Responder for the post-hoc analysis of leg pain is a participant whose leg pain score improved by 30% or more. Odds ratio (versus Sham) from a logistic regression model estimating the probability of being a responder with treatment group as a factor and baseline score as a covariate. An odds ratio > 1 means participants in test groups are more likely to be responders compared to those in the Sham group. Participants with no baseline leg pain were excluded from the MITT analyses for both planned and post-hoc tests

### Primary outcome – COPM performance

The commonly reported COPM functional activities in this study are detailed in Additional Table 3. The improvement in COPM-P was statistically significantly greater in both test groups compared to Sham, at both Week 8 and Week 12 (*p* < 0.001) in the change score analysis. Size of this difference was 1-point or greater. The percentage of participants achieving MCID (responders achieving 2-point change) at week 8 was 61% for Program 1 and 60% for Program 2, compared to 21% for Sham (absolute risk difference of approximately 40%). Hence, the odds of achieving MCID in either test group was approximately 4.8 times the odds of achieving MCID in Sham (*p* < 0.005). At week 12 follow-up, the responder rates remained significantly greater in both test groups, with odds ratios of 3.3 for Program 1 and 4.4 for Program 2 versus Sham (*p* < 0.05).

### Secondary outcomes

#### COPM satisfaction

The COPM-S results were similar to that of COPM-P, with both change score and responder (MCID) analyses showing statistically significantly greater improvement in both test groups compared to Sham, at both Week 8 and Week 12 (*p* < 0.005). Size of this difference was 1-point or greater. The odds ratios for achieving MCID were 8.80 for Program 1 versus Sham (*p* < 0.0001) and 7.16 for Program 2 versus Sham (*p* = 0.0004) at week 8, and 4.80 for Program 1 versus Sham (*p* = 0.0027) and 4.86 for Program 2 versus Sham (*p* = 0.0029) at week 12.

#### Leg pain ITT analysis

The improvement in leg pain change score was statistically significantly greater in both test groups compared to Sham, at both week 8 (*p* < 0.01) and week 12 (*p* < 0.05). Size of this difference was greater than 1-point. The odds ratios for achieving MCID were 2.8 for Program 1 versus Sham (*p* = 0.0523) and 4.2 for Program 2 versus Sham (*p* = 0.0113). At week 12 there were no significant differences between the groups (*p* > 0.05).

#### Leg pain MITT responder analysis

Fourteen participants did not report leg pain (as it was not a mandatory inclusion criterion) at baseline (5 participants each from Programs 1&2, and 4 participants from Sham). These 14 participants were excluded from the MITT analysis, which was considered more clinically meaningful (for all other outcomes ITT analysis was equal to MITT analysis). The participants excluded from the MITT population were equally distributed across treatment groups and did not experience any worsening of their pain during the study (pain scores remained at zero throughout). The MITT analysis showed that the relative benefit in either test group was greater compared to Sham. The odds ratios for achieving MCID were 2.91 for Program 1 versus Sham (*p* = 0.0426) and 4.03 for Program 2 versus Sham (*p* = 0.0174). At week 12 there was no significant difference for Program 1 versus Sham (*p* > 0.05).

#### Leg pain post-hoc responder analysis

As stated, for the post-hoc responder analysis for leg pain, the definition of a responder was: a participant whose pain score improved by at least 30% from baseline (instead of the conventional 2-point absolute change). For both ITT and MITT analyses, the percentage of participants achieving a 30% reduction in pain after 8 weeks of device use was statistically significantly greater in Program 1 and Program 2 compared to Sham (*p* < 0.05). The odds ratios for achieving MCID were 3.36 for Program 1 versus Sham (*p* = 0.016) and 3.03 for Program 2 versus Sham (*p* = 0.0257) for the post-hoc ITT analysis, and 4.05 for Program 1 versus Sham (*p* = 0.0075) and 3.25 for Program 2 versus Sham (*p* = 0.0247) for the post-hoc MITT analysis. At week 12 there was no significant difference for Program 1 versus Sham (*p* > 0.05).

#### Leg symptoms score

The improvement in overall leg symptoms scores were statistically significantly greater in both test groups compared to Sham, at both Week 8 and Week 12. The size of the difference at Week 8 was greater than 4 points (*p* < 0.001) (-10.17 in Program 1, -10.49 in Program 2 compared to -5.95 in Sham). The treatment benefit decreased by Week 12 but was still statistically significant (*p* < 0.05). No responder analysis was carried out for this outcome as MCID have not been established. Analysis of the individual components of the leg symptoms score is given in Additional Table 4.

#### Blood flow volume and intensity

In groups using active devices, ankle blood flow volume increased approximately 3-fold (mean of 2.58 at baseline to 7.51 during use). Similarly, blood flow intensity increased 3-fold in the active groups (mean of 223 at baseline to 730 during use). There were no notable changes for either measure in the Sham group.

## Discussion

Although the participants in this study did not necessarily have a formal diagnosis, the reported leg symptoms of pain and cramps, and sensations of aching, heaviness, and tiredness are usually consistent with impairment in blood circulation relating to PVDs such as CVD or PAD (although a diagnostic correlation is unclear) [3–6, 10]. Such conditions are common yet underdiagnosed among community-dwelling older adults [54, 55]. Up to 80% of the population suffer from at least a mild form of CVD and over 230 million people worldwide are affected by PAD causing a significant global health and economic impact [33, 56, 57]. The vascular system degenerates with age indicating an increased risk of severe cardiovascular events such as stroke and myocardial infarction [56, 57], which gets worsened by declining physical activity and deterioration of functional QoL [58]. When vascular diseases remain underdiagnosed and the emanating symptoms receive little medical attention, alternative home-based ‘over the counter’ treatments such as ‘foot NMES’ that provide effective early intervention for the management of leg symptoms become particularly important [32, 33, 59, 60]. Moreover, recent studies have shown that referral and participation in recommended treatments for PAD (such as supervised exercise therapy (SET)) is very low and therefore they remain highly underutilised due to challenges in awareness, access, and implementation [61, 62].

Foot NMES devices can be purchased over the counter without prescription and are designed for self-use at home without supervision. This pragmatic study was therefore conducted in a real-world setting, where the participants self-administered the treatment at home and were not required to make any alteration to their medication, diet, or exercise. This study has, to our knowledge, for the first time provided real-world data informing the applicability of foot NMES in community-dwelling older adults for the management of leg pain and other leg symptoms, and for improving their everyday functional performance. The results will therefore inform clinical practice.

Improvements in both COPM performance and satisfaction were highly clinically significant, with the percentage of responders nearly three times greater in the two Revitive^®^ test groups compared to Sham. Similarly, the odds of achieving MCID for leg pain were nearly three-times greater in the two Revitive^®^ test groups compared to Sham. The significant reduction in overall leg symptoms scores further supported these findings. The study demonstrated that these foot NMES devices are safe to use at home without supervision and that compliance was high, which corroborates earlier research [32–34]. Overall, the significant improvements and clinically relevant changes in the subjective outcomes indicate that the benefits delivered by foot NMES in real-world use are clinically relevant (results met MCID for COPM and leg pain) and were meaningful to the study participants. The study also demonstrated a sizeable ‘placebo effect’ for most outcomes. However, this is not unusual for a sham-controlled study given that placebo effect may exist with ‘perceived intervention’, mainly due to ‘expectation’ [63, 64]. On the other hand, the placebo effect together with high compliance showed that participants were adequately blinded to the Sham. Notwithstanding the placebo effect, the real treatment effect was statistically significantly greater and clinically meaningful.

This study had various strengths and some limitations. To the authors’ knowledge this is the first study of its kind on this important topic. The study featured a sham control and was adequately statistically powered. The primary outcome was ‘patient-centred’, evaluating self-reported functions that were important to the participants. Compliance with the intervention was high and the dropout rates were low. One limitation was that the study was only participant-blinded, the assessor blinding would have been problematic for reasons previously identified. The follow-up was short, but many of the outcomes were still significant at 12 weeks.

## Conclusions

The home-based foot NMES therapy using Revitive^®^ Medic© Program 1 and Revitive^®^ Program 2 over an 8-week period significantly improved self-reported function, reduced leg pain, relieved leg symptoms, and increased ankle blood flow (during treatment) compared to Sham. Compliance with the intervention was high (> 92% in the test groups) indicating that the device was well tolerated and was sufficiently easy to use and manage. No device-related adverse events were reported, which demonstrated the high degree of safety. It is anticipated that with continued foot NMES use more sustained and greater treatment effects may be achieved; however, this should be investigated by further studies with potentially longer follow-up, which per se was beyond the scope of this study.

### Electronic supplementary material

Below is the link to the electronic supplementary material.

Supplementary Material 1

## Data Availability

Anonymised summary data will be available from the corresponding author upon reasonable request.

## Abbreviations

AE: Adverse event
ANCOVA: Analysis of covariance
COPM-P: Canadian occupational performance measure – performance
COPM-S: Canadian occupational performance measure – satisfaction
CVD: Chronic venous disease
ITT: Intention to treat
HSETECDA: University of Hertfordshire Health, Science, Engineering and Technology Ethics Committee with Delegated Authority
MCID: Minimal clinically important difference
MITT: Modified intention to treat
NMES: Neuromuscular electrical stimulation
NPRS: Numerical pain rating scale
PP: Per protocol
PAD: Peripheral arterial disease
PVD: Peripheral vascular disease
QoL: Quality of life
SAE: Serious adverse event
SET: Supervised exercise therapy
UAE: Unrelated adverse event
