# Supplementary material for: Benefits of home-based foot neuromuscular electrical stimulation on self-reported function, leg pain and other leg symptoms among community-dwelling older adults: a sham-controlled randomised clinical trial
Source: BMC Geriatr. 2024 Aug 14;24:683. doi: 10.1186/s12877-024-05271-z (PMC11323382; doi:10.1186/s12877-024-05271-z)
Supplement: Supplementary file 1 — Supplementary Material 1 [file 12877_2024_5271_MOESM1_ESM.docx]

Benefits of home-based foot neuromuscular electrical stimulation on self-reported function, leg pain and other leg symptoms among community-dwelling older adults: A sham-controlled randomised clinical trial.

# Additional data

## Additional Table 1: Participant self-reported comorbidities – ITT population.

| **Comorbidity** | **Number of people reporting** | | |
| --- | --- | --- | --- |
|  | **Program 1**  **N = 41** | **Program 2**  **N = 42** | **Sham**  **N = 44** |
| Coronary artery disease or other cardiovascular (hypertension, hyperlipidemia, arrhythmia, etc.) | 19 | 20 | 17 |
| Type II Diabetes | 3 | 7 | 5 |
| Past history of cancer | 6 | 8 | 5 |
| Osteoarthritis hips / knees | 2 | 12 | 4 |
| Mechanical low back pain | 10 | 2 | 8 |
| Asthma / COPD | 3 | 3 | 2 |
| Thyroid disease | 2 | 3 | 3 |
| Enlarged prostate | 2 | 1 | 3 |

## Additional table 2: Mean (SD) / median percentage change from baseline – ITT population.

| **Outcome and the timepoint** | | **Statistic** | | **Program 1 (N = 41)** | | **Program 2 (N = 42)** | | **Sham (N = 44)** | |
| --- | --- | --- | --- | --- | --- | --- | --- | --- | --- |
| **COPM-P** | |  | |  | |  | |  | |
| Week 8 | | n | | 41 | | 39 | | 39 | |
|  | | Missing | | 0 | | 3 | | 5 | |
|  | | Mean | | 49.39 | | 55.28 | | 19.74 | |
|  | | SD | | 34.549 | | 47.960 | | 23.629 | |
|  | | Median | | 46.15 | | 47.37 | | 14.67 | |
|  | | | | | | | | | |
| Week 12 | | n | | 38 | | 39 | | 37 | |
|  | | Missing | | 3 | | 3 | | 7 | |
|  | | Mean | | 44.97 | | 50.34 | | 18.67 | |
|  | | SD | | 31.861 | | 44.449 | | 23.405 | |
|  | | Median | | 39.32 | | 41.67 | | 8.33 | |
| **COPM-S** | |  | |  | |  | |  | |
| Week 8 | | n | | 41 | | 39 | | 39 | |
|  | | Missing | | 0 | | 3 | | 5 | |
|  | | Mean | | 74.99 | | 67.71 | | 28.16 | |
|  | | SD | | 70.830 | | 66.379 | | 54.490 | |
|  | | Median | | 54.04 | | 62.79 | | 8.94 | |
|  | | | | | | | | | |
| Week 12 | | n | | 38 | | 39 | | 37 | |
|  | | Missing | | 3 | | 3 | | 7 | |
|  | | Mean | | 65.31 | | 62.00 | | 27.30 | |
|  | | SD | | 58.517 | | 54.972 | | 54.441 | |
|  | | Median | | 56.09 | | 58.82 | | 8.33 | |
|  | |  | |  | |  | |  | |
| **Leg pain** | |  |  |  |  |  |  |  |  |
| Week 8 | | n | | 36 | | 34 | | 35 | |
|  | | Missing | | 5 | | 8 | | 9 | |
|  | | Mean | | -59.33 | | -53.43 | | -34.02 | |
|  | | SD | | 35.762 | | 33.708 | | 34.521 | |
|  | | Median | | -65.48 | | -42.86 | | -28.57 | |
|  | | | | | | | | | |
| Week 12 | | n | | 33 | | 34 | | 33 | |
|  | | Missing | | 8 | | 8 | | 11 | |
|  | | Mean | | -47.66 | | -49.45 | | -26.99 | |
|  | | SD | | 37.660 | | 39.301 | | 33.134 | |
|  | | Median | | -43.75 | | -43.30 | | -16.67 | |
| **Overall leg symptoms score** | |  | |  | |  | |  | |
| Week 8 | | n | | 41 | | 39 | | 39 | |
|  | | Missing | | 0 | | 3 | | 5 | |
|  | | Mean | | -66.81 | | -70.15 | | -39.31 | |
|  | | SD | | 35.812 | | 27.482 | | 42.207 | |
|  | | Median | | -80.61 | | -76.27 | | -32.65 | |
|  | | | | | | | | | |
| Week 12 | | n | | 38 | | 39 | | 37 | |
|  | | Missing | | 3 | | 3 | | 7 | |
|  | | Mean | | -63.50 | | -60.17 | | -40.52 | |
|  | | SD | | 34.811 | | 40.006 | | 43.338 | |
|  | | Median | | -75.06 | | -70.96 | | -43.87 | |
|  | |  | |  | |  | |  | |
| **Overall leg symptoms score – individual symptoms** | |  | |  | |  | |  | |
|  | |  | |  | |  | |  | |
| **Feeling of heaviness in legs** | |  | |  | |  | |  | |
|  | |  | |  | |  | |  | |
| Week 8 | | n | | 35 | | 30 | | 29 | |
|  | | Missing | | 6 | | 12 | | 15 | |
|  | | Mean | | -61.86 | | -69.14 | | -42.32 | |
|  | | SD | | 36.365 | | 33.614 | | 46.250 | |
|  | | Median | | -62.50 | | -74.64 | | -40.00 | |
|  | |  | |  | |  | |  | |
| Week 12 | | n | | 33 | | 30 | | 27 | |
|  | | Missing | | 8 | | 12 | | 17 | |
|  | | Mean | | -59.95 | | -50.48 | | -48.08 | |
|  | | SD | | 39.114 | | 75.285 | | 41.313 | |
|  | | Median | | -66.67 | | -72.32 | | -42.86 | |
|  | |  | |  | |  | |  | |
| **Feeling of tiredness in legs** | |  | |  | |  | |  | |
|  | |  | |  | |  | |  | |
| Week 8 | | n | | 37 | | 30 | | 28 | |
|  | | Missing | | 4 | | 12 | | 16 | |
|  | | Mean | | -62.13 | | -69.48 | | -34.56 | |
|  | | SD | | 44.299 | | 33.346 | | 42.938 | |
|  | | Median | | -71.43 | | -73.66 | | -26.79 | |
| Week 12 | | n | | 34 | | 30 | | 27 | |
|  | | Missing | | 7 | | 12 | | 17 | |
|  | | Mean | | -62.79 | | -66.04 | | -40.14 | |
|  | | SD | | 42.224 | | 47.764 | | 46.866 | |
|  | | Median | | -81.17 | | -75.89 | | -40.00 | |
|  | |  | |  | |  | |  | |
| **Feeling of aching in legs** | |  | |  | |  | |  | |
|  | |  | |  | |  | |  | |
| Week 8 | | n | | 35 | | 33 | | 35 | |
|  | | Missing | | 6 | | 9 | | 9 | |
|  | | Mean | | -67.26 | | -70.98 | | -42.75 | |
|  | | SD | | 42.172 | | 29.161 | | 39.198 | |
|  | | Median | | -86.81 | | -79.59 | | -33.33 | |
|  | |  | |  | |  | |  | |
| Week 12 | | n | | 32 | | 33 | | 33 | |
|  | | Missing | | 9 | | 9 | | 11 | |
|  | | Mean | | -54.92 | | -59.74 | | -40.06 | |
|  | | SD | | 45.629 | | 39.930 | | 37.309 | |
|  | | Median | | -69.64 | | -65.63 | | -42.86 | |
|  | |  | |  | |  | |  | |
| **Feeling of cramps in legs** | |  | |  | |  | |  | |
|  | |  | |  | |  | |  | |
| Week 8 | | n | | 34 | | 34 | | 30 | |
|  | | Missing | | 7 | | 8 | | 14 | |
|  | | Mean | | -77.80 | | -77.61 | | -46.48 | |
|  | | SD | | 37.505 | | 31.179 | | 50.899 | |
|  | | Median | | -100.00 | | -90.00 | | -57.14 | |
|  | |  | |  | |  | |  | |
| Week 12 | | n | | 31 | | 34 | | 29 | |
|  | | Missing | | 10 | | 8 | | 15 | |
|  | | Mean | | -69.35 | | -66.39 | | -40.96 | |
|  | | SD | | 37.851 | | 43.256 | | 63.115 | |
|  | | Median | | -83.33 | | -77.92 | | -50.00 | |
|  | |  | |  | |  | |  | |

Footnotes: N = number of participants in the analysis population, n = number of participants in the outcome subset. SD = standard deviation. COPM-P&S measured from 0 (worst) to 10 (best). Leg pain measured from 0 (least pain) to 10 (worst pain). Some of the missing counts in leg pain are due to baseline pain values of 0 and so percentage change calculation is not possible. Overall leg symptoms score can range from 0 (least symptoms) to 40 (worst symptoms). Individual leg symptoms score can range from 0 (least symptoms) to 10 (worst symptoms).

## Additional Table 3: COPM activities most commonly reported by the participants – ITT population.

| **Activity** | **Number of people reporting** | | |
| --- | --- | --- | --- |
|  | **Program 1**  **N = 41** | **Program 2**  **N = 42** | **Sham**  **N = 44** |
| Sleeping | 27 | 34 | 31 |
| Walking | 27 | 27 | 31 |
| Standing | 31 | 14 | 14 |
| Sitting | 14 | 24 | 24 |
| Stair climbing | 20 | 14 | 27 |
| General daily activities | 7 | 7 | 4 |

## Additional Table 4: Overall leg symptoms score individual symptoms analysis – ITT population.

| **Symptom and the timepoint** | | **Statistic** | | **Program 1 (N = 41)** | | **Program 2 (N = 42)** | | **Sham (N = 44)** | |
| --- | --- | --- | --- | --- | --- | --- | --- | --- | --- |
| **Feeling of heaviness in legs** | |  | |  | |  | |  | |
| Week 8 | | n | | 41 | | 39 | | 39 | |
|  | | Mean | | -2.72 | | -1.98 | | -1.49 | |
|  | | Median | | -1.86 | | -1.71 | | -1.00 | |
|  | | p value (vs. Sham) | | 0.0123 | | 0.3344 | |  | |
|  | | | | | | | | | |
| Week 12 | | n | | 38 | | 39 | | 37 | |
|  | | Mean | | -2.68 | | -1.59 | | -1.51 | |
|  | | Median | | -2.14 | | -1.71 | | -0.57 | |
|  | | p value (vs. Sham) | | 0.0249 | | 0.4207 | |  | |
|  | |  | |  | |  | |  | |
| **Feeling of tiredness in legs** | |  | |  | |  | |  | |
| Week 8 | | n | | 41 | | 39 | | 39 | |
|  | | Mean | | -2.74 | | -2.17 | | -1.46 | |
|  | | Median | | -2.29 | | -1.36 | | 0.00 | |
|  | | p value (vs. Sham) | | 0.0060 | | 0.0548 | |  | |
|  | | | | | | | | | |
| Week 12 | | n | | 38 | | 39 | | 37 | |
|  | | Mean | | -2.66 | | -2.05 | | -1.61 | |
|  | | Median | | -2.00 | | -1.43 | | 0.00 | |
|  | | p value (vs. Sham) | | 0.0188 | | 0.1068 | |  | |
|  | |  | |  | |  | |  | |
| **Feeling of aching in legs** | |  |  |  |  |  |  |  |  |
| Week 8 | | n | | 41 | | 39 | | 39 | |
|  | | Mean | | -3.36 | | -3.08 | | -1.98 | |
|  | | Median | | -4.29 | | -3.00 | | -1.00 | |
|  | | p value (vs. Sham) | | 0.0234 | | 0.0238 | |  | |
|  | | | | | | | | | |
| Week 12 | | n | | 38 | | 39 | | 37 | |
|  | | Mean | | -2.51 | | -2.50 | | -1.87 | |
|  | | Median | | -2.43 | | -2.00 | | -1.00 | |
|  | | p value (vs. Sham) | | 0.2698 | | 0.1304 | |  | |
|  | |  | |  | |  | |  | |
| **Feeling of cramps in legs** | |  | |  | |  | |  | |
| Week 8 | | n | | 41 | | 39 | | 39 | |
|  | | Mean | | -2.32 | | -2.61 | | -1.29 | |
|  | | Median | | -2.43 | | -2.29 | | -0.57 | |
|  | | p value (vs. Sham) | | 0.0034 | | 0.0018 | |  | |
|  | | | | | | | | | |
| Week 12 | | n | | 38 | | 39 | | 37 | |
|  | | Mean | | -2.03 | | -2.37 | | -1.05 | |
|  | | Median | | -1.71 | | -2.29 | | -0.64 | |
|  | | p value (vs. Sham) | | 0.0191 | | 0.0020 | |  | |

Footnotes: N = number of participants in the analysis population, n = number of participants in the symptom subset. Symptoms score can range from 0 (least symptoms) to 10 (worst symptoms). p value (vs. Sham) from a Wilcoxon rank sum test compared to Sham with missing data imputed using Baseline Observation Carried Forward.
